# Semen Handling in South American Camelids: State of the Art

**DOI:** 10.3389/fvets.2020.586858

**Published:** 2020-11-06

**Authors:** Jane M. Morrell, Maria Celina Abraham

**Affiliations:** Clinical Sciences, Swedish University of Agricultural Sciences, Uppsala, Sweden

**Keywords:** viscous seminal plasma, ovulation inducing factor, dribble ejaculators, single layer centrifugation, epididymal spermatozoa, sperm preservation

## Abstract

Reproductive biotechnologies such as artificial insemination could be very useful for South American camelids, allowing widespread use of semen from breeding males with desirable genetics. However, artificial insemination is not widely employed in these species and is considered to have low overall efficiency. This is due in part to incomplete knowledge about the physiology of conception in these species, and also to challenges presented by semen collection and handling. Several recent reviews have centered on female camelid reproduction; therefore, in this review, the focus is on semen handling. Various semen collection methods are presented. Different methods of reducing seminal viscosity are compared, such as needling, enzyme treatment, and colloid centrifugation. Use of enzymes remains controversial because of widely differing results among research groups. Colloid centrifugation, particularly single layer centrifugation, has proved to be successful in facilitating development of sperm handling techniques in dromedary camels, and has also been used with llama semen. Therefore, protocols for colloid centrifugation of alpaca semen could be developed in the future.

## Introduction

Although artificial insemination (AI) is common in other domestic livestock, the use of this reproductive biotechnology in camelids is limited, but especially so for South American camelids (SACs) (1). In any species, many production traits can be improved by careful selective breeding stock with desirable genetics (2). Fiber quality in SACs is no exception: it can be improved by selective breeding using animals with high quality fiber (3). In theory, AI could be very useful for improving fiber quality in SACs by allowing more widespread breeding from genetically elite individuals, but protocols for this reproductive biotechnology have not been optimized in these species.

Knowledge about reproductive physiology in SACs is currently incomplete (4), thus hindering the development of protocols for AI. Thus, factors such as the optimum timing of AI relative to ovulation, the number of spermatozoa required, and the site of semen deposition need to be defined for the different species. In addition, techniques for semen preservation require optimization (5). Moreover, SACs are induced ovulators, with an ovulation-inducing factor (recently identified as nerve growth factor) in seminal plasma being the main ovulation-inducing agent (6). Therefore, if AI is used, it may be necessary to induce ovulation, either by injecting hormones, mating to a vasectomized male, or inseminating seminal plasma at the same time as the spermatozoa. The optimal timing of ovulation induction relative to semen deposition is not known.

The viscous character of camelid semen is one of the main limiting factors in the development of AI (7). Other important factors limiting reproduction in alpacas are their low overall fertility, which is due both to low sperm production and a high incidence of pseudo pregnancy or early embryo mortality (8), and the physiological capacity of females that usually have no more than four offspring throughout their life (9).

As an alternative to AI, embryo transfer has been attempted in SACs (10). Successful within-species embryo transfer was achieved in both llamas (11–14) and alpacas (15, 16). Cross-species embryo transfer between SACs was also accomplished with *in vivo* generated embryos (12, 17, 18). Embryos have been produced *in vitro* from llamas (19), and recently also from alpacas (20). However, it is not clear whether offspring resulted from transfer of such *in vitro* derived embryos.

A recent review on SAC (5) summarized the available literature on timing of AI, sperm numbers, deposition site, and how ovulation was induced, together with the outcome. The purpose of the present review, therefore, is to examine the problems associated with semen handling in SACs and look at possible solutions that have been successful or partially successful in a related species, the dromedary camel.

## Semen Collection

All reproductive biotechnologies require a source of good quality semen. Semen collection in SACs is challenging, not least because mating takes place in sternal recumbency and is of long duration (21). Semen can be collected by different methods, including an artificial vagina, vaginal sponges, and electro-ejaculation, as reviewed recently (22). Creating a urethral fistula provides access to small quantities of spermatozoa without seminal plasma but is not practical as a regular supply of spermatozoa for AI. There may be ethical and welfare aspects related to some of these methods. Thus, for example, anesthesia is required, or is mandatory in some countries, for electroejaculation, since it can cause extreme muscular contractions. Surgical alterations to provide a source of spermatozoa without seminal plasma would not be considered ethical in many countries, and may present welfare issues. The advantages and disadvantages of the different methods are summarized in Table 1.

**Table 1:** Advantages and disadvantages of various semen collection methods in alpacas [modified from (22)].

**Technique**	**Advantages**	**Disadvantages**
Artificial vagina	Relatively clean “physiological” samples; No contact with female reproductive tract secretions.	Males must be trained; cannot be used for isolated semen collections in untrained individuals.
Electroejaculation	Any mature individual.	Anesthesia required, restricting number of collections from each individual; ejaculate may not be complete or may be contaminated with urine^*^.
Post-mating aspiration	No training of males needed.	Semen contaminated with secretions from female tract, bacteria etc. Not universally acceptable as source of semen for AI. Restraint of females may cause stress.
Epididymal spermatozoa (castration; post mortem)	By-product from slaughterhouses or from normal husbandry technique.	Only one sample from each male unless hemicastration performed.
Urethral fistula	Provides spermatozoa without viscous seminal plasma.	Small numbers of spermatozoa collected. Fistula must be kept open^*^.
Others (intravaginal sacs or condom, vaginal sponges, aspiration after mating)	No training of males needed.	Insertion of intravaginal devices not easy and stressful if animals not accustomed to handling. Devices may hamper intromission; Devices or aspiration can cause injury. Contamination with vaginal secretions, bacteria.

* *may not be considered acceptable in some countries on welfare grounds*.

An artificial vagina is the preferred method for collecting samples in alpacas and llamas (23). The samples collected are not usually contaminated with urine, which can be a problem with electroejaculation (21), and contains the contributions from the accessory sex glands in the physiological proportions. Once the male has mounted a female or a phantom, the erect penis is introduced into the artificial vagina and ejaculation occurs into a sterilized collection vessel. In the dromedary camel, using a phantom instead of a live female enabled a sample to be collected without contamination (24). In a study comparing semen collection by artificial vagina and electroejaculation in llamas, the proportion of successful semen collections was reported to be greater when using an electro-ejaculator than an artificial vagina (25). Semen volume was greater and both sperm motility and membrane integrity were higher in samples collected by electroejaculation than by artificial vagina. However, the method may not be suitable for repeated use on the same males in the field and requires anesthesia because the intense muscular contractions produced can be painful (4).

Aspiration of semen from the reproductive tract of a mated female, or extraction from vaginal sponges or condoms, results in samples that may be contaminated by other secretions and cells (21), resulting in poor sperm survival. Sperm recovery may be stressful for females that are not accustomed to being handled (22) and the method is not practical for most alpaca husbandry systems. Although this method is a source of ejaculated spermatozoa, aspiration from the vagina for transfer into other females is not desirable for biosecurity [(26), cited in (22)].

## Semen Characteristics

As previously mentioned, camelid semen is highly viscous (7), forming a gel immediately after ejaculation. Although the gel is rich in glycosaminoglycans (GAG), this component is not considered to cause the viscosity, since treatment with enzymes specific for GAGs does not reduce the viscosity appreciably whereas treatment with proteases does (27). The viscosity is, instead, attributed to the high mucin content as detailed in Kershaw-Young and Maxwell (27). More recent studies on the protein components of camelid seminal plasma have been mostly directed toward characterizing the ovulation-inducing factor in the seminal plasma, which is beyond the scope of this review [e.g., (28)].

The spermatozoa are retained within this gelatinous mass, making it difficult to separate the spermatozoa from the seminal plasma. Thus, simple techniques, such as evaluating sperm concentration or sperm motility by conventional means, are challenging. The viscous seminal plasma might be an adaptation to retain the spermatozoa in the female's reproductive tract until ovulation, which occurs ~30 h after copulation in the alpaca (29). Sperm motility in this gelatinous mass is oscillatory rather than progressive (30).

Camelids ejaculate in fractions throughout the whole copulation (“dribble ejaculation”); sometimes only gelatinous seminal plasma is ejaculated without any spermatozoa (31). The volume of the ejaculate varies, e.g., from 0.4 to 4.3 mL, and the average seminal plasma volume was 1.5 ± 0.1 mL (32). The semen is usually opalescent to milky white, depending on sperm concentration (33), which ranges from 62 to 750 ×10^6^ spermatozoa/mL, with an average motility of 68–85% (21).

## Semen Handling

The high viscosity of the seminal plasma creates difficulties in sperm assessment and handling. The spermatozoa are trapped within the gel and show an oscillatory motility pattern rather than a progressive pattern (32). It is almost impossible to make smears from this material, and the gel appears to prevent dyes from penetrating the sperm membrane for evaluation of membrane integrity. The presence of seminal plasma may also hinder penetration of cryoprotectants (27). Certainly, alpaca spermatozoa freeze poorly using currently available protocols, and no offspring have been produced following AI with frozen semen (5). However, there are reports of successful freezing of llama spermatozoa (19), with offspring born after AI.

Several methods to reduce seminal plasma viscosity and release trapped spermatozoa have been attempted. These include needling, pipetting, sperm washing, addition of enzymes, and colloid centrifugation. Needling and pipetting, i.e., repeated aspiration of semen through a needle or a pipette, respectively, may help liquefaction but sperm quality tends to be reduced, possibly due to physical damage or by release of reactive oxygen species that may subsequently affect sperm membranes and chromatin. Prior extension of the semen sample with a buffered semen extender, followed by gentle pipetting during incubation, was shown to liquefy semen from dromedary camels (34). Removal of the freed spermatozoa from the seminal plasma is needed as the gel tends to reappear with time, trapping the spermatozoa once more. Colloid centrifugation (see sperm selection) was more effective for removing spermatozoa from liquefied seminal plasma than centrifugation without a colloid, since it allowed the spermatozoa to be completely separated from the seminal plasma (34).

The gel fraction of the seminal plasma is thought to be due to the presence of proteins such as mucins. In an attempt to reduce the viscosity of the semen, researchers have tested various proteases with varying degrees of success (summarized in Table 2). However, the use of enzymes is controversial since they may damage spermatozoa (10). One explanation for the differing results presented by various researchers could be the considerable variation in viscosity among camelid ejaculates. The degree of viscosity affects the concentration of enzyme needed, or the time required for it to act. However, exposing spermatozoa to any enzymes could be expected to have an adverse effect on their membranes. Thus, the affected spermatozoa might still be able to function in IVF shortly after enzyme treatment, or if the spermatozoa are rapidly removed from the media containing the enzymes, whereas they are unable to function if preserved for subsequent use in AI.

**Table 2:** Summary of various studies on enzyme treatment of semen from South American camelids.

**Enzyme**	**Species**	**Effect**	**Source**
Trypsin	Alpaca	Sperm motility ↓	(35)
Trypsin, collagenase, hyaluronidase, and fibrinolysin	Alpaca and llama	Not possible to obtain progressive motility	(36)
Trypsin	Alpaca and llama	Detached heads	(37)
Collagenase at 0.5, 1.0, 2.0, and 4.0 mg/mL	Alpaca	Toxic to sperm	(38)
1 mg/ml collagenase	Llama	Spermatozoa are not adversely affected	(19, 39)
Hyaluronidase, chondroitinase ABC, and keratinase) and proteases (papain and proteinase K)	Alpaca	Papain was most promising in reducing thread formation	(40)
Papain	Alpaca	Does not reduce sperm motility, viability, DNA integrity or acrosome integrity	(41)
200 or 600 units/mL catalase	Alpaca	Spermatozoa are not adversely affected	(5)

*↓, decreased*.

Colloid centrifugation is a so-called biomimetic sperm preparation technique, whereby the sperm selection that occurs in the female reproductive tract is mimicked *in vitro*. Briefly, in the female reproductive tract, motile spermatozoa migrate away from seminal plasma; non-motile spermatozoa are removed by back-flow (42). Spermatozoa that are free of seminal plasma interact with the uterine and oviductal epithelial cells, and are thought to be retained in the crypts of the uterotubal junction where they initiate the changes that occur during capacitation. They are released when ovulation occurs, and locate the oocyte for fertilization.

Several biomimetic techniques are available that simulate this *in vivo* sperm selection (43). These include migration techniques, e.g., “swim-up,” filtration, magnetic activated cell sorting, and colloid centrifugation. Colloids can be used as a density gradient, i.e., with two or more layers of colloid of different densities, or with only one layer of colloid (Single Layer Centrifugation, SLC). This technique has been used for sperm selection in many different species [reviewed by (43)]. Since spermatozoa are separated from seminal plasma as well as selecting the robust spermatozoa (44), the method could be beneficial in extracting camelid spermatozoa from seminal plasma. Of the different selection techniques, colloid centrifugation looks to be quite promising, at least for llama spermatozoa (10, 19), and is now used regularly when preparing dromedary camel semen for reproductive biotechnologies (45). Use of a low density gradient made from a colloid designed for human spermatozoa was also reported for preparing alpaca spermatozoa (5). It should be noted that in the case of the low density colloid, the purpose of the colloid is merely to separate the spermatozoa from the seminal plasma rather than to select robust spermatozoa from the rest of the ejaculate (46). Higher density colloids are used for selection of robust spermatozoa that are more likely to be capable of achieving fertilization.

A comparison of swim-up and colloid centrifugation of llama spermatozoa (after treatment of the ejaculate with collagenase) concluded that colloid centrifugation was the method of choice for preparing spermatozoa (39). In studies on dromedary camel semen that had been subjected to gentle pipetting, i.e., without enzyme treatment, Malo et al. (34) showed that sperm quality parameters and *in vitro* fertilization ability of spermatozoa were improved by SLC compared with simple sperm washing. The colloid separated live motile spermatozoa from seminal plasma, dead cells, debris, and extender. The same researchers were able to cryopreserve dromedary camel sperm samples (47–49) and obtain offspring after AI with the thawed samples. Since colloid centrifugation seems to represent a reliable, repeatable, and relatively simple way of extracting camelid spermatozoa without damage, it could provide the way forward when working with alpaca semen.

### Semen Extenders

A variety of different extenders have been used for camelid semen, as reviewed recently (5). Evaluating which extenders function best for each species is problematic because it is not known which methods for evaluating sperm quality are reliable as indicators of fertility in SACs. Once a method for AI in these species has been optimized, it may be possible to relate sperm quality in different extenders to fertility, thus facilitating development of optimized extenders for these species.

### Pregnancy Rates

The success of any one semen handling method compared to another is usually measured in terms of pregnancy rate and births. Although some pregnancies and live births have been achieved following AI in SACs, the success rate is low (5). In an alpaca study, 1 out of 42 inseminated females gave birth (4). A 21.7% success rate was reported for a study on llamas (50). Such low success rates imply that the methods used are still sub-optimal. A comparison of the different methodologies is provided by (5). It would be interesting to see pregnancy rates from the use of sperm samples prepared by colloid centrifugation without the use of enzymes, since pregnancy rates are higher in other species following colloid selection, e.g., stallion (51).

## Current Challenges and Possible Solutions

The protocols currently available for semen collection, extracting spermatozoa from the ejaculate, and sperm preservation are not effective for alpaca spermatozoa.

As presented here, there are indications that colloid centrifugation presents a practical solution for viscosity reduction in llamas and dromedary camel semen, either following brief enzyme treatment or after pipetting in the presence of buffer. Therefore, optimizing these protocols for alpaca semen is strongly recommended. In the meantime, initial studies on developing preservation or cryopreservation protocols could be carried out with epididymal spermatozoa, which can be obtained either as a byproduct of castration or from slaughterhouse material. Although they represent a useful source of spermatozoa for describing the characteristics of alpaca spermatozoa (52), or for testing extenders and freezing protocols [(52), personal communication] they have limitations for more general use in AI. For obvious reasons, regular sperm collections from the same male are not possible and therefore biological replicates of experiments are not feasible, and it can be difficult to harvest the spermatozoa without blood or cellular contamination. Furthermore, it is not known whether extenders and preservation protocols derived using epididymal spermatozoa are relevant for working with ejaculated spermatozoa. However, this material could be a useful starting point in the development of sperm preservation protocols.

## Further Considerations

Developing sperm handling procedures is only a first step in developing AI. If AI is to become a reality in SACs, timing of ovulation relative to AI, sperm numbers deposited, and the site of semen deposition have to be established. The timing of ovulation induction relative to insemination should be investigated, e.g., with the help of ultrasound to pinpoint ovulation. Obtaining a consistent supply of ejaculated spermatozoa that can be manipulated is essential to carrying out studies that are reliable and repeatable. One point is clear; there is still plenty of opportunity for research in these interesting species.

## Author Contributions

JM and MA researched the literature. JM drafted the review. MA checked it. Both authors contributed to the article and approved the submitted version.

## Conflict of Interest

JM is the inventor and one of the patent holders of the colloids mentioned in this article. The remaining author declares that the research was conducted in the absence of any commercial or financial relationships that could be construed as a potential conflict of interest.
